# Supplementary material for: Effects of Deoxynivalenol and Fumonisins Fed in Combination to Beef Cattle: Immunotoxicity and Gene Expression
Source: Toxins (Basel). 2021 Oct 10;13(10):714. doi: 10.3390/toxins13100714 (PMC8541374; doi:10.3390/toxins13100714)
Supplement: Supplementary file 1 [file toxins-13-00714-s001.zip › toxins-1377613-supp.pdf]

# Supplementary Materials: Effects of Deoxynivalenol and Fumonisins Fed in Combination on Beef Cattle: Immunotoxicity and Gene Expression

**Heaven L. Roberts, Massimo Bionaz, Duo Jiang, Barbara Doupovec, Johannes Faas, Charles T. Estill, Dian Schatzmayr and Jennifer M. Duringer**

## 1. Evaluation of Phagocytosis and Oxidative Burst in Beef Cattle Whole Blood

All centrifuge steps were completed at room temperature. Samples were covered with foil during incubations and storage to protect them from light. All homogenizations were performed by pipetting gently. All supernatant was discarded by gently discarding the top layer (avoiding disturbance of the cell pellet), and briefly touching the rim of the tube to a Kimwipe (Kimberly Clark, city, state, USA) to remove residual fluid. Gibco calcium and magnesium-free phosphate buffered saline (Thermo-Fisher Scientific; PBS) was used for reagent prep and in place of all fluorochromes as needed (e.g. when no antibody was added for negative or FMO controls, etc.). Oxidative burst kit modifications include replacing RPMI/FBS-based assay buffer with 1X PBS. Additionally, the lysis buffer that came with the oxidative burst kit was poorly functioning after 1-2 weeks of storage at 4°C in method development runs in our lab; thus, lysis steps from the pHrodo kit were used in this assay as well.

### *1.1. Solution preparation (used in both assays)*

Two mL of Buffer B was added to the vial containing the lyophilized product in each kit to resuspend the pHrodo™ BioParticles® conjugate. Each vial was vortexed for 1 minute and sonicated for 5 minutes until all the fluorescent particles were homogenously dispersed. BioParticles solution was aliquotted and samples stored at -20°C. Solution was thawed via body heat from hands (protected from light) on the day of use, then kept on ice ~20 minutes before adding to samples. Diluted dihydrorhodamine 123 (DHR 123) reagent was prepared on the day of sampling by adding 0.25 μL DHR 123 stock solution + 250 μL 1X PBS (sufficient for 12 samples + controls). Phorbol 12-myristate 13-acetate (PMA) was prepared by adding 1.7 μL to 1000 μL 1X PBS. *Note that this was higher than the PMA concentration in the kit's instructions (up to 500 nM is traditionally used, the kit called for 200 nM). A higher PMA concentration was adopted after troubleshooting revealed more consistent activation in pooled control samples with adoption of altered lysis steps, per the manufacturer's guidance*. Antibodies were diluted on the day of sampling using 0.5 μL of each antibody stock solution in 100 μL 1X PBS. Diluted antibodies were stored at room temperature, protected from light and used within 1 hour of preparation. Hoechst stock solution (2 mg/mL) was stored at -20°C and diluted on the day of use by adding 1 μL of Hoechst stock solution to 5 mL of 1X PBS. 1.6% neutral buffered formalin solution was prepared in 1X PBS from 16% stock solution stored at room temperature. Lysis buffers from the pHrodo kit were kept at room temperature prior to use.

### *1.2. Sample preparation (used in both assays)*

Whole blood was collected into sodium heparin tubes, rocked to homogenize and transported to the lab at room temperature. A 2.0 mL microcentrifuge tube was prepared with 100 μL of blood from each steer (pooled control, n=6); sample was pipetted gently to homogenize. For all samples, 100 μL of blood from each individual animal was added to the 2.0 mL microcentrifuge tube.

### *1.3. Bioparticle addition (phagocytosis assay)*

BioParticles (20 μL) was added to each sample and appropriate controls, then homogenized. Tubes were placed in a 37°C water or bead bath for 30 minutes. All samples were then transferred to ice for 10 minutes to cool before lysis.

### *1.4. DHR and PMA addition (oxidative burst assay)*

Ten µL of the working solution of DHR 123 was added to samples and controls, then homogenized. Samples were incubated at 37°C for 15 minutes. 25 µL of working solution of PMA was added, then the mixture homogenized by gently pipetting. Samples were incubated at 37°C for 45 minutes. All samples were placed on ice for 10 minutes to cool before lysis.

### *1.5. Erythrocyte lysis (used in both assays)*

100 µL of pHrodo kit Lysis Buffer A was added to blood samples and homogenized. Tubes were incubated for 5 minutes at room temperature. One mL of pHrodo kit Lysis Buffer B was added, then samples were homogenized. Tubes were incubated for 5 minutes at room temperature, then centrifuged at 500 × *g* for 5 minutes and the supernatant discarded.

### *1.6. Antibody addition (used in both assays)*

The cell pellet remaining after erythrocyte lysis was resuspended in 100 µL of each primary antibody solution (or 1X PBS, as appropriate). Samples were incubated for 30 minutes at room temperature, then centrifuged at 1000 × *g* for 5 minutes; the supernatant was discarded. 100 µL of each secondary antibody and 100 µL Hoechst solution was added to the samples and controls, homogenized, then incubated for 20 minutes at room temperature. Samples were centrifuged at 1000 × *g* for 5 minutes and the supernatant discarded.

### *1.7. Fixation (used in both assays)*

The cell pellet of all samples and controls was resuspended in 500 µL 1.6% formalin solution (1 part 16% stock, 9 parts 1X PBS). Fluorochromes were stable during testing in formalin for up to 48 hours at 4°C; however, we found it best to rinse the samples prior to storage. Samples were stored for 20 minutes at room temperature for fixation prior to rinsing. To rinse a sample of formalin, it was centrifuged at 1000 × *g* for 5 minutes, then the supernatant was discarded. All samples were resuspended in 250 µL of 1X PBS, then centrifuged again and resuspended in 250 µL of 1X PBS. 200 µL was transferred to each well of a 96 well flat-bottomed plate through 70 µm mesh netting just prior to flow cytometric analysis.

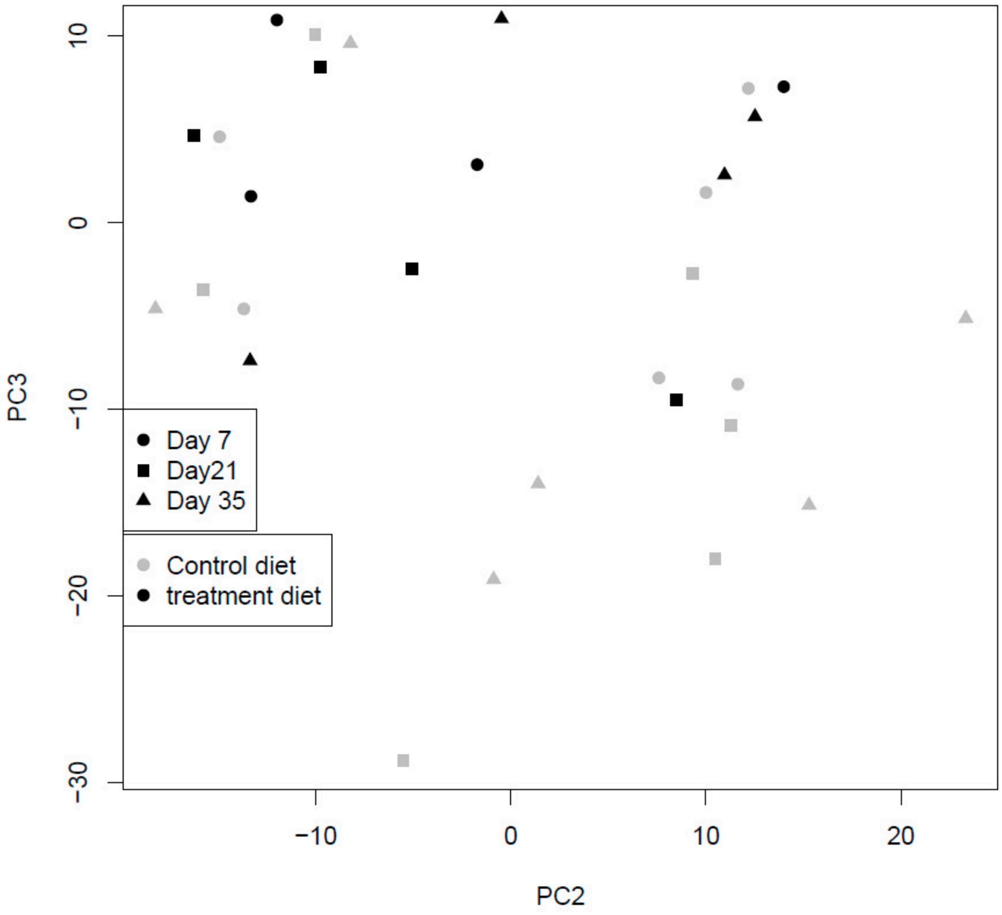


**Figure S1.** Scatterplot of second (PC2) and third (PC3) principal components of the RNA-Scheme 2. Example gating diagram of CD4 and CD8 lymphocyte profiles used to evaluate the effects of deoxynivalenol and fumonisins fed in combination on beef cattle. Data shown from a single animal at day 21 of the trial (Treatment period lasted three weeks, followed by a two week clearance period)

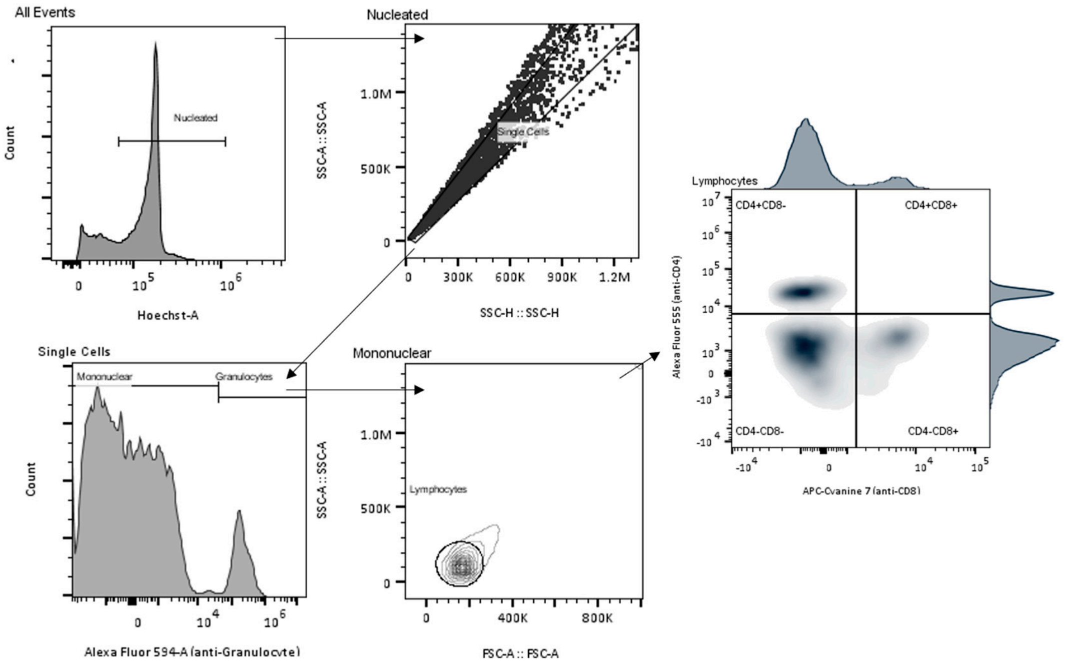


Figure S2. Flow cytometric panel used to quantify phagocytotic capacity and leukocyte populations in beef cattle fed deoxynivalenol and fumonisins in combination.

**Table S1.** Flow cytometric panel used to quantify phagocytotic capacity and leukocyte populations in beef cattle fed deoxynivalenol and fumonisins in combination.[1.]

| Primary Antibody | Fluorochrome / Secondary Antibody | Population | Laser (nm) | Filter |
|---|---|---|---|---|
| - | pHrodo[2] | Phagocytic Cells | 488 | 525/40 |
| - | Hoechst[2] | Nucleated Cells | 405 | 450/45 |
| CH138A (mouse IgM)[3] | Alexa Fluor 594 (goat anti-mouse IgM)[2] | Granulocytes | 561 | 610/20 |
| IL11A (mouse IgG2a)[3] | Alexa Fluor 555 (goat anti-mouse IgG2a)[2] | $CD4^+$ lymphocytes | 561 | 585/42 |
| CACT80C (mouse IgG1)[3] | APC/Cy7 (goat anti-mouse IgG1)[4] | $CD8^+$ lymphocytes | 638 | 780/60 |

[1]Treatment period lasted three weeks, followed by a two week clearance period. [2]Thermo-Fisher Scientific, Waltham, MA, USA; [3]Washington State Monoclonal Antibody Center, Pullman, WA, USA; [4]Southern Biotech, Birmingham, AL, USA.

**Table S2.** Flow cytometric panel to quantify oxidative burst capacity in beef cattle fed deoxynivalenol and fumonisins in combination.[1.]

| Primary Antibody | Fluorochrome | Population | Laser (nm) | Filter |
|---|---|---|---|---|
| - | DHR-123[2] | Oxidizing Cells | 405 | 525/40 |
| - | Hoechst[3] | Nucleated Cells | 405 | 450/45 |
| CH138A (Mouse IgM)[4] | Alexa Fluor 594 (goat anti-mouse IgM)[3] | Granulocytes | 561 | 610/20 |

[1]Treatment period lasted three weeks, followed by a two week clearance period. [2]Cayman Chemical, Ann Arbor, Michigan, USA; [3]Thermo-Fisher Scientific, Waltham, MA, USA; [4]Washington State Monoclonal Antibody Center, Pullman, WA, USA.

**Table S3.** Differentially expressed genes in beef cattle fed deoxynivalenol and fumonisins in combination.[1.]

| See attached excel file (Supp_Tables_S3S4). |
|---|

[1]Treatment period lasted three weeks, followed by a two week clearance period.

**Table S4.** Genes expressed in beef cattle fed deoxynivalenol and fumonisins in combination.[1.]

| See attached excel file (Supp_Tables_ S3S4). |
|---|

[1]Treatment period lasted three weeks, followed by a two week clearance period.

**Table S5.** DAVID settings for analysis of differentially expressed genes in beef cattle fed deoxynivalenol and fumonisins in combination.[1.]

| Option Group | Option |
|---|---|
| Annotation | Count Threshold: 2 |
| | EASE Score Threshold: 0.10 |
| Clustering | Classification Stringency: Medium |
| | Kappa Similarity - Similarity Term Overlap: 3 |
| | Kappa Similarity - Similarity Threshold: 0.5 |
| | Classification - Initial Group Membership: 3 |
| | Classification - Final Group Membership: 3 |
| | Classification - Multiple Linkage Threshold: 0.5 |
| | EASE Score Enrichment Threshold: 1.0 |

[1]Treatment period lasted three weeks, followed by a two week clearance period.

**Table S6–S14.** DAVID gene expression clustering from beef cattle fed deoxynivalenol and fumonisins in combination.[1.]

| See attached excel file (Supp_Tables_S6S14). |
|---|

[1]Treatment period lasted three weeks, followed by a two week clearance period.

**Table S15–S23.** DAVID terms from beef cattle fed deoxynivalenol and fumonisins in combination.[1.]

| See attached excel file (Supp_Tables_S15S23). |
|---|

[1]Treatment period lasted three weeks, followed by a two week clearance period.

**Table S24–S26.** Sorted DIA terms from beef cattle fed deoxynivalenol and fumonisins in combination.[1.]

| See attached excel file (Supp_Tables_S24S26). |
|---|

[1]Treatment period lasted three weeks, followed by a two week clearance period.

**Table 27.** Mycotoxin analysis of total mixed ration used to evaluate the effects of deoxynivalenol and fumonisins fed in combination on beef cattle.[1,2.]

| | Fumonisins (B1, B2, B3) (ppm) | Deoxynivalenol (ppb) | Nivalenol (ppb) | Aflatoxins (B1, B2, G1, G2) (ppb) | Ochratoxin A (ppb) | Acetyldeoxynivalenol, fusarenon X, T-2 toxin, HT-2 toxin, neosolaniol, diacetoxyscirpenol (ppb) | Zearelenone (ppb) |
|---|---|---|---|---|---|---|---|
| *TMR Week One* | | | | | | | |
| Control | 0.4 | 268.4 | 165.0 | <LOD | <LOD | <LOD | <LOD |
| Treatment | 3.0 | 1352.5 | 105.1 | <LOD | <LOD | <LOD | 137.8 |
| *TMR Week Two* | | | | | | | |
| Control | 0.2 | <LOD | <LOD | <LOD | <LOD | <LOD | <LOD |
| Treatment | 10.6 | 2045.2 | 121.9 | <LOD | <LOD | <LOD | 114.5 |
| *TMR Week Three* | | | | | | | |
| Control | <LOD | 183.9 | <LOD | <LOD | <LOD | <LOD | <LOD |
| Treatment | 3.0 | 2087.6 | 116.3 | <LOD | <LOD | <LOD | <LOD |

[1]Treatment period lasted three weeks, followed by a two week clearance period (weeks 4 and 5).
[2]LOD: aflatoxin B1 (1.3 ppb); aflatoxin B2 (1.2 ppb); aflatoxin G1 (1.1 ppb); aflatoxin G2 (1.6 ppb);

fumonisin B1,B2,B3 (0.1 ppm); ochratoxin A (1.1 ppb); deoxynivalenol, acetyldeoxynivalenol, fusarenon X, nivalenol, T-2 toxin, HT-2 toxin, neosolaniol, diacetoxyscirpernol (100 ppb); zearalenone (51.7 ppb).

**Table S28.** Dietary analysis of total mixed ration used to evaluate the effects of deoxynivalenol and fumonisins fed in combination on beef cattle.[1,2.]

| | **Dry Matter (% as fed)** | **CP/ACP (%DM)** | **SP (%CP)** | **ADF (%DM)** | **aNDF (%DM)** | **Lignin (%DM)** | **NFC (%DM)** | **Starch (%DM)** | **EE (%DM)** | **Ash (%DM)** | **TDN (%DM)** |
|---|---|---|---|---|---|---|---|---|---|---|---|
| *TMR Week One* | | | | | | | | | | | |
| Control | 88.4 | 10.5 | 26 | 12.4 | 20.4 | 1.0 | 61.5 | 48.3 | 3.1 | 4.55 | 81 |
| Treatment | 89.3 | 10.0 | 23 | 10.9 | 20.3 | 1.0 | 62 | 47.9 | 2.5 | 5.13 | 80 |
| *TMR Week Three* | | | | | | | | | | | |
| Control | 89.3 | 10.0 | 23 | 10.9 | 20.3 | 1.0 | 62 | 47.9 | 2.5 | 5.13 | 80 |
| Treatment | 88.9 | 10.5 | 23 | 10.6 | 19.5 | 1.4 | 61.3 | 47.4 | 3.2 | 5.46 | 80 |
| *TMR Week Four* | | | | | | | | | | | |
| Control | 88.7 | 10.1 | 25 | 11.9 | 21.1 | 1.4 | 60.8 | 46.0 | 2.5 | 5.52 | 78 |
| Treatment | 88.6 | 9.9 | 26 | 11.7 | 20.5 | 1.2 | 61.3 | 46.9 | 2.7 | 5.61 | 79 |
| *TMR Week Five* | | | | | | | | | | | |
| Control | 88.7 | 9.3 | 29 | 14.4 | 24.2 | 1.7 | 58.6 | 43.3 | 2.3 | 5.48 | 77 |
| Treatment | 89.7 | 9.2 | 25 | 12.6 | 23.2 | 1.9 | 59.7 | 45.5 | 2.4 | 5.5 | 77 |
| *Flaked Corn*[3] | 85.8 | 9.4 | 16 | 2.9 | 8.7 | 1.1 | 77.4 | 74.9 | 2.9 | 1.6 | 87 |
| *Ground Control Corn* | 86.8 | 9.4 | 22 | 3.5 | 10.1 | 1.1 | 74.5 | 68.3 | 3.9 | 2.08 | 87 |
| *Ground Treatment Corn* | 84.5 | 8.3 | 24 | 3.3 | 8.1 | 1.1 | 77.9 | 72.8 | 4.3 | 1.44 | 89 |
| *Fescue Hay* | 92.7 | 5.1 | 38 | 43.1 | 67.2 | 5.2 | 21.7 | 0.7 | 1.0 | 5.03 | 57 |

[1]Treatment period lasted three weeks, followed by a two week clearance period (weeks 4 and 5). DM = Dry Matter; CP = Crude Protein; ACP = Adjusted Crude Protein; SP = Soluble Protein; ADF = Acid Detergent Fiber; and = Neutral Detergent Fiber; NFC = Non-fiber Carbohydrates; EE = Ether Extract (fat); TDN = Total Digestible Nutrients. [2]Previously published in Duringer et al., (2020) *World Mycotoxin Journal* 13, 533–543. [3]Flaked corn fed only during animal arrival and transition.
